# Simplified abductor pollicis longus suspension interposition arthroplasty for thumb carpometacarpal joint osteoarthritis

**DOI:** 10.1007/s00068-020-01577-w

**Published:** 2020-12-24

**Authors:** Anna Lena Sander, Clara Friederike Buhrmann, Katharina Sommer, Johannes Frank

**Affiliations:** Department of Trauma, Hand and Reconstructive Surgery, University Hospital Frankfurt, Theodor-Stern-Kai 7, 60590 Frankfurt am Main, Germany

**Keywords:** Thumb carpometacarpal joint osteoarthritis, Abductor pollicis longus, Suspension, RegJoint™, Interposition, Arthroplasty

## Abstract

**Purpose:**

The primary treatment goals for advanced-stage thumb carpometacarpal (CMC) joint osteoarthritis are complete pain relief and restoration of thumb strength. The purpose of the present study was to introduce a variation of the abductor pollicis longus (APL) suspension arthroplasty using a single looping of a radial slip from the APL tendon around the flexor carpi radialis (FCR) tendon combined with RegJoint™ interposition and to determine its efficacy in the treatment of thumb CMC joint osteoarthritis.

**Methods:**

Between 2015 and 2017, 21 patients were included. The average age was 60.8 years (range 48–79). The mean follow-up was 27.7 months (range 8–50). Evaluation included pain, radial and palmar abduction, tip pinch and grip strength, and Disabilities of the Arm, Shoulder, and Hand (DASH) score.

**Results:**

Pain averaged 0.3 (range 0–4) at rest and 1.4 (range 0–4) on exertion. The radial and palmar abduction were 97% and 99% compared to the contralateral side. The tip pinch and grip strength were 4.1 kg (range 3–6.5) and 22 kg (range 13.3–40), respectively. The DASH score accounted for 18.5 (range 0.8–41.7).

**Conclusion:**

The modified APL suspension interposition arthroplasty was an efficient and simplified option for the treatment of thumb CMC joint osteoarthritis, with results comparable or better than other published procedures. The APL suspension technique was easy to perform avoiding difficult bone tunneling and incision of the FCR tendon. The RegJoint™ interposition as spacer prevented impingement of the first metacarpal base on the second metacarpal base or the trapezoid bone.

## Introduction

Surgical treatment of advanced-stage thumb carpometacarpal (CMC) joint osteoarthritis aims at relieving pain while restoring thumb stability and strength [1]. Numerous procedures have been described, which can be categorised in trapeziectomy, suspension and/or interposition arthroplasty, total joint arthroplasty, and CMC joint fusion (arthrodesis) [2–9]. Although a recent systemic review by the Cochrane Collaboration was unable to demonstrate that any technique confers a benefit over another, suspension arthroplasty is preferred by surgeons concerned that proximal migration of the first metacarpal and radial displacement forces may impair function [10–13]. While good long-term results have been reported in terms of pain relief and restoration of function, several of the suspension arthroplasties require extensive bone tunneling and/or looping of the suspension [14,15]. Therefore, we developed a simplified variation of the abductor pollicis longus (APL) suspension technique described by Lundborg combined with bioabsorbable poly-l/d-lactide joint implant (RegJoint™; Scaffdex, Tampere, Finland) interposition [8,16,17]. The purpose of this study was to describe this technique and to determine its efficacy in the treatment of thumb CMC joint osteoarthritis.

## Patients and methods

### Patients

Approval from the institutional review board of the medical faculty (GN235/17) was obtained prior to performing this study. A retrospective analysis was performed on a cohort of patients who underwent surgery for thumb CMC joint osteoarthritis between 2015 and 2017. The inclusion criteria in this study were (1) thumb CMC joint osteoarthritis, (2) severe pain limiting the patient activities of daily living, (3) Eaton and Littler grade II-IV on preoperative X-rays, and (4) APL suspension interposition arthroplasty as chosen treatment, with the indication for surgery determined primarily by clinical findings and not radiological grade [3]. The exclusion criteria were (1) CMC arthrodesis and (2) relevant concomitant pathologies of the wrist and/or hand. Twenty-nine patients with thumb CMC joint osteoarthritis were identified. Two patients were excluded due to the exclusion criteria. Six patients were lost to follow-up due to the following reasons: (1) the patient did not want to participate in the study, (2) the contact information was no longer correct, and (3) the patient was not able to participate in the study due to long journey.

A total of 21 patients could be examined for follow-up. The average age was 60.8 years (range 48–79). The ratio of male to female patients was 1:3.2. The dominant hand was affected in 43%. The mean period of follow-up was 27.7 months (range 8–50) (Table 1).

**Table 1:** Patients

Number of patients	21
Age (years)	60.8 (48–79)
Gender (m:w)	1:3.2
Dominant hand (%)	43
Follow-up (months)	27.7 (8–50)

### Clinical assessment

Pain was assessed using the visual analogue scale (VAS 1–10). Pain experienced during manual use was defined as exertional.

Range of motion (ROM) was measured with a standard goniometer. The ROM included radial and palmar abduction. The results were reported as a percentage of the contralateral side.

Tip pinch strength was determined by a Baseline® Lite Hydraulic Pinch Gauge (Fabrication Enterprises, Inc., White Plains, New York, USA) and grip strength by a Baseline® Hydraulic Hand Dynamometer (Fabrication Enterprises, Inc., White Plains, New York, USA) at level 2. Each hand was measured three times and the mean values were calculated.

The Disabilities of the Arm, Shoulder, and Hand (DASH) score (0: no limitation, 100: maximum limitation) was used to evaluate functional outcome [18,19].

The pattern of returning to work was recorded in those patients who were still working.

Complications during the first six months were documented at the follow-up assessment.

### Surgical technique

**Fig. 1:**
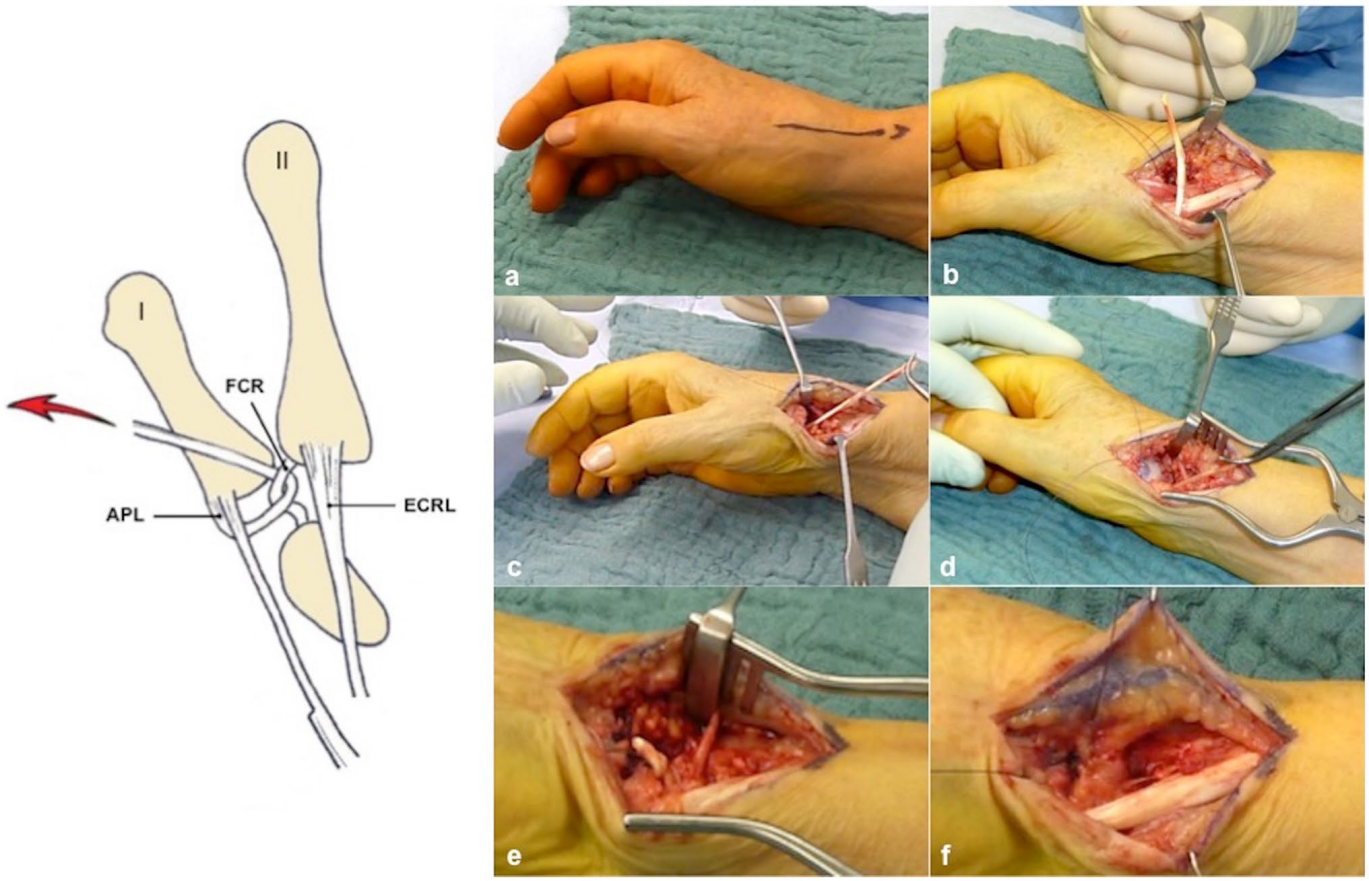
**a** Longitudinal skin incision of 3–4 cm over the thumb CMC joint between the APL and EPL tendon. **b** Dissection of one substantial radial slip of the APL tendon distally based on the first metacarpal base. **c** Looping of the APL tendon slip around the FCR tendon and suture to itself under vertical traction. **d** Interposition of the RegJoint™ between the sling and the first metacarpal base. **e** Suture of the dorsal capsule of the trapezial space. **f** Protection of the superficial branches of the radial nerve with a prepared fat flap

The operation was performed under regional or general anaesthesia. A pneumatic tourniquet was routinely used with a pressure of 280 mmHg. A longitudinal skin incision of 3–4 cm was made over the thumb CMC joint between the APL tendon and the extensor pollicis longus (EPL) tendon (Fig. 1a). The superficial branches of the radial nerve and the radial artery were identified and protected. Dissection was extended down to the capsule of the thumb CMC joint. A longitudinal capsulotomy was performed. The trapezium and osteophytes at the first and second metacarpal base were completely removed. The flexor carpi radialis (FCR) tendon was protected during the trapeziectomy. One substantial radial slip of the APL tendon, left attached on the first metacarpal base, was harvested through the same incision. By elevation of the skin and subcutaneous tissues, including the superficial branches of the radial nerve, and division of the extensor retinaculum of the first compartment, it was possible to harvest the entire length of the APL tendon back to the musculotendinous junction, avoiding further scars on the forearm. If the APL tendon was not composed of two or more slips, but by a large insertion, a radial slip of the single tendon was dissected (Fig. 1b). The harvested APL tendon slip was passed under the remaining APL tendon slip. Afterwards, it was looped around the FCR tendon without incision of the FCR tendon and sutured to itself under vertical traction (Fig. 1c). A RegJoint™ was used as small spacer between the sling and the first metacarpal base without contact to cancellous bone to prevent impingement of the first metacarpal base on the second metacarpal base or the trapezoid bone. The fixation was performed at the volar capsule (Fig. 1d). The dorsal capsule of the trapezial space was closed (Fig. 1e). A prepared fat flap was used to protect the superficial branches of the radial nerve (Fig. 1f). Haemostasis and skin suture completed the procedure. A cast was applied to the thumb with the CMC joint in slight abduction, immobilising the wrist and the CMC and metacarpophalangeal joints of the thumb and leaving the interphalangeal joint free. After 2 weeks, a thumb orthosis was recommended for 4 weeks. Full weight-bearing was allowed after a total period of 12 weeks.

## Results

Postoperative pain symptoms averaged 0.3 (range 0–4) at rest and 1.4 (range 0–4) on exertion.

The average ROM was 97% for radial abduction and 99% for palmar abduction compared to the contralateral side.

The mean postoperative tip pinch and grip strength reached 4.1 kg (range 3–6.5) and 22 kg (range 13.3–40), respectively.

At the final examination, the mean DASH score averaged 18.5 (range 0.8–41.7).

Of the working patients in this study, 53% returned to their original occupations and 47% to their original occupations with job modification (Table 2).

**Table 2:** Results

Pain	
At rest	0.3 (0–4)
On exertion	1.4 (0–4)
ROM (% contralateral)	
Radial abduction	97
Palmar abduction	99
Tip pinch strength (kg)	4.1 (3–6.5)
Grip strength (kg)	22 (13.3–40)
DASH score	18.5 (0.8–41.7)
Occupation (%)	
Original	53
Original with job modification	47

Two cases of temporary dysaesthesia of the dorsoradial region of the thumb and FCR tendinosis were recorded. No case of tendon (slip) rupture, adverse soft tissue reaction, or complex regional pain syndrome (CRPS) occurred.

## Discussion

Complete pain relief is one of the primary treatment goals for advanced-stage thumb CMC joint osteoarthritis [12]. Junior et al. evaluated the results of ten patients using a technique of APL interposition arthroplasty with temporary Kirschner wire fixation followed up over a mean period of 41.6 months. The VAS measured 7.77 before surgery and 2.04 after surgery [20]. Nanno et al. investigated the clinical results of 16 patients who were treated with a modified Thompson procedure with a mean follow-up of 25 months. The VAS score markedly improved from 8.64 to 1.2 [9,21]. Rab et al. focused on the long-term results of 20 patients who underwent APL suspension arthroplasty similar to the Lundborg method with a mean follow-up of 23.1 months. The pain at rest was 6.7 ± 1.9 preoperatively versus 1.1 ± 1.7 postoperatively [8,22,23]. Singer and Kandel assessed the outcome of 20 APL suspension interposition arthroplasties with an average follow-up of 24 months. The mean VAS improved from 6.2 ± 1.54 to 1.1 ± 0.97 [24]. In the present study, the results on the VAS were comparable to better. Postoperative pain symptoms were determined with 0.3 (range 0–4) at rest and 1.4 (range 0–4) on exertion.

Restoration of near full mobility with stability of the ray is a relevant indicator for the constitution of the suspension. In the study of Junior et al., radial and palmar abduction presented significant improvements after the operation from 30.2° to 40.3° and 32° to 39.5° [20]. Nanno et al. evaluated the average radial and palmar abduction with 30.9° and 36.5° before surgery and 54.1° and 48.9° after surgery. The radial abduction after surgery was significantly better than before surgery [21]. In the study by Rab et al., the ROM of radial abduction after arthroplasty was 63.4° ± 14.3° versus 60.3° ± 12.7° for palmar abduction [22]. Sirotakova et al. determined the efficacy of a variation of the APL suspension arthroplasty of 104 trapeziectomies in 74 consecutive patients at 12 months after surgery. Radial abduction increased from 47° before surgery to 53° after surgery (91% of the contralateral side) and palmar abduction from 44° to 47° (87%), respectively [12]. Our results were congruent or better with radial and palmar abduction ROM of 97% and 99% when compared with the contralateral side.

Effective surgical treatment of thumb CMC joint osteoarthritis further aims to restore thumb strength [12]. In the study of Junior et al., the tip pinch strength presented significant improvement after surgery from 3.3 to 5.5 kg. The grip strength decreased significantly from 18.2 to 11.3 kg [20]. Nanno et al. showed that average pinch tip and grip strength markedly improved from 3.43 kg and 13.3 kg before surgery to 4.61 kg and 18.8 kg after surgery [21]. Rab et al. evaluated tip pinch and grip strength with 6.2 ± 2.8 kg and 23.9 ± 9.7 kg, respectively [22]. Sirotakova et al. reported tip pinch strength with 2.6 kg preoperatively and 4 kg postoperatively, compared to grip strength with 13 kg and 18 kg [12]. In the present study, the results were similar with a tip pinch and grip strength of 4.1 kg (range 3–6.5) and 22 kg (range 13.3–40).

An outcome measure, which reflects the impact on function, is a key component in the assessment of treatment success. In the study by Nanno et al., the Quick DASH score markedly improved from 43.6 before surgery to 13.6 after surgery [21]. Rab et al. evaluated a DASH score of 20.1 ± 15.1 [22]. Rocchi et al. tested the looping of a slip from the APL tendon around the first intermetacarpal ligament at 42 patients who were followed up to one year. Subjective assessment totalled a DASH score of 43.3 preoperatively. After surgery, DASH score progressively lowered to 25.5 three months postoperatively, then to 19.1 after six months, and reached 14.5 after one year [15]. The average Quick DASH score of Singer and Kandel markedly decreased from 47.6 ± 8.81 to 13.6 ± 5.46 [24]. Our results lied in between with a DASH score of 18.5 (range 0.8–41.7).

Given that most surgical techniques have a high success rate, perhaps the most important part of articles describing procedures should be a discussion of complications. Junior et al. evaluated four patients with temporary dysaesthesia of the dorsoradial region of the thumb and one patient with early loosening of the Kirschner wires [20]. In the study by Rab et al., no complications occurred [22]. Rocchi et al. recorded one case of keloid and two cases of temporary dysaesthesia of the dorsoradial region of the thumb [15]. Sirotakova et al. assessed three patients with CRPS, one patient with a localised soft tissue infection and temporary paraesthesia of the dorsoradial region of the thumb, and four patients with temporary pulling discomfort of the FCR tendon. Eight thumbs showed ongoing pain, which could be mimicked by lateral pressure on the first metacarpal base from the radial side. It was believed that the cause was an impingement of an osteophyte on the ulnovolar surface of the first metacarpal base on the second metacarpal base or the trapezoid bone. Pain relief was achieved by excision of the osteophyte and interposition of a palmaris tendon anchovy between the metacarpal bases [12]. In the present study, the complication rate was comparable with two cases of temporary dysaesthesia of the dorsoradial region of the thumb and FCR tendinosis, but without cases of tendon (slip) ruptures, adverse soft tissue reactions, or CRPS. The RegJoint™ interposition was validated as there was no clinical evidence of impingement, which confirms the algorithm of Sirotakova et al. [12].

Some limitations must be considered for our study. First, the study design was retrospective. Second, follow-up was only available for a relatively small number of patients, which, however, is consistent with current literature. Third, pain perception, and DASH score before treatment were missing. Even though this study contributes to currently data, future research should involve prospective clinical and radiological outcome studies.

## Conclusion

In conclusion, the modified APL suspension interposition arthroplasty was an efficient and simplified option for the treatment of thumb CMC joint osteoarthritis, with results comparable or better than other published procedures. The suspension technique was easy to perform based on a single looping of a radial slip from the APL tendon around the FCR tendon while avoiding difficult bone tunneling and incision of the FCR tendon. The RegJoint™ interposition as spacer prevented impingement of the first metacarpal base on the second metacarpal base or the trapezoid bone.
